# Genomic Delineation of Zoonotic Origins of *Clostridium difficile*

**DOI:** 10.3389/fpubh.2019.00164

**Published:** 2019-06-20

**Authors:** Daniel R. Knight, Thomas V. Riley

**Affiliations:** ^1^Medical, Molecular, and Forensic Sciences, Murdoch University, Perth, WA, Australia; ^2^School of Medical and Health Sciences, Edith Cowan University, Joondalup, WA, Australia; ^3^School of Biomedical Sciences, The University of Western Australia, Nedlands, WA, Australia; ^4^PathWest Laboratory Medicine, Department of Microbiology, Nedlands, WA, Australia

**Keywords:** evolution, transmission, *Clostridium difficile*, one health, livestock, zoonosis

## Abstract

*Clostridium difficile* is toxin-producing antimicrobial resistant (AMR) enteropathogen historically associated with diarrhea and pseudomembranous colitis in hospitalized patients. In recent years, there have been dramatic increases in the incidence and severity of *C. difficile* infection (CDI), and associated morbidity and mortality, in both healthcare and community settings. *C. difficile* is an ancient and diverse species that displays a sympatric lifestyle, establishing itself in a range of ecological niches external to the healthcare system. These sources/reservoirs include food, water, soil, and over a dozen animal species, in particular, livestock such as pigs and cattle. In a manner analogous to human infection, excessive antimicrobial exposure, particularly to cephalosporins, is driving the expansion of *C. difficile* in livestock populations worldwide. Subsequent spore contamination of meat, vegetables grown in soil containing animal feces, agricultural by-products such as compost and manure, and the environment in general (households, lawns, and public spaces) is contributing to a persistent community source/reservoir of *C. difficile* and the insidious rise of CDI in the community. The whole-genome sequencing era continues to redefine our view of this complex pathogen. The application of high-resolution microbial genomics in a One Health framework (encompassing clinical, veterinary, and environment derived datasets) is the optimal paradigm for advancing our understanding of CDI in humans and animals. This approach has begun to yield critical insights into the genetic diversity, evolution, AMR, and zoonotic potential of *C. difficile*. In Europe, North America, and Australia, microevolutionary analysis of the *C. difficile* core genome shows strains common to humans and animals (livestock or companion animals) do not form distinct populations but share a recent evolutionary history. Moreover, for *C. difficile* sequence type 11 and PCR ribotypes 078 and 014, major lineages of One Health importance, this approach has substantiated inter-species clonal transmission between animals and humans. These findings indicate either a zoonosis or anthroponosis. Moreover, they challenge the existing paradigm and the long-held misconception that CDI is primarily a healthcare-associated infection. In this article, evolutionary, and zoonotic aspects of CDI are discussed, including the anthropomorphic factors that contribute to the spread of *C. difficile* from the farm to the community.

## Introduction

Last year was the 40th anniversary of the publication in 1978 of a series of papers from several research groups that provided proof that *Clostridium difficile* caused pseudomembranous colitis (1–4). While the spectrum of gastrointestinal disease caused by *C. difficile* has broadened significantly since then, for much of those 40 years *C. difficile* was thought of as causing disease almost exclusively within high-risk hospitalized patient populations (5). In evolutionary terms, 40 years is a negligible length of time. The *Clostridia* are an ancient prokaryotic lineage, estimated to have diverged from the bacterial domain 2.34 Ga (billion years) ago around the time when concentrations of molecular oxygen in the atmosphere began to increase (6). With the advances of next-generation sequencing, the taxonomy of the *Clostridia* is currently undergoing a major revision. Indeed, given the significant differences between *C. difficile* and some other pathogenic clostridia, it has been proposed that it be renamed *Clostridioides difficile* (7). While this has caused some angst in the *C. difficile* community, both names are currently viewed as being “validly published” and therefore acceptable (8).

In recent years, the vast majority of emerging or re-emerging infections have been vector-borne or zoonoses—animal diseases that are transmissible to humans (9). Most attention has focused on viral infections because of highly publicized outbreaks; SARS, avian influenza, and Ebola. However, disease associated with *C. difficile* infection (CDI) has killed more people worldwide in the last 15 years than all these viral infections combined, around 30,000 per year in the USA alone according to the CDC (10). CDI should always have been considered a zoonosis, either direct or indirect. In some definitions of zoonoses, non-human animal hosts play an essential role in maintaining the infection in nature and humans are only incidental hosts. In CDI, all animals (human and non-human) are likely hosts; the wide variety of animals from which *C. difficile* has already been isolated suggests this (11).

What then is the natural history of CDI following exposure to *C. difficile*? *C. difficile* is ubiquitous in the environment. *C. difficile* colonizes the gastrointestinal tracts of all animals during the neonatal period, multiplies, and is excreted, but cannot/does not compete well when other bacterial species start to colonize. The exact timing of this change is not clear, but it is probably linked to changes in diet in babies, i.e., weaning. Through a process known as colonization resistance, a well-developed microbiota provides protection against overgrowth of *C. difficile* by inhibiting germination, vegetative growth, and toxin production (12). In human and non-human animals, antimicrobial exposure creates an environment that could be thought of as mimicking the neonatal gut—characterized by an underdeveloped microbiota and consequently reduced or absent colonization resistance. In such a compromised host gut, *C. difficile* spores rapidly germinate and begin to produce potent cytotoxins (toxin A and toxin B) which cause extensive colonic inflammation and epithelial tissue damage, the net effect being a rapid fluid loss into the intestinal lumen which manifests as diarrhea (13). Some strains also produce a binary toxin, an ADP-ribosyltransferase that causes actin cytoskeletal disruption, and is associated with more severe CDI, a higher case-fatality rate and refractory disease (14).

When those antimicrobials were cephalosporins in the 1980s and 90s, antimicrobials to which *C. difficile* is intrinsically resistant, there was an expansion of CDI in hospitals that continues today. Since the 1990s in North America, cephalosporins have been licensed for use in food animals. There has been an amplification of *C. difficile* in food animals since then, with subsequent contamination of meat, and vegetables grown in soil containing animal feces. In some animals such as piglets, there is overt disease with significant impact on industry. “Animal” strains of *C. difficile* are now infecting humans. *C. difficile* ribotype (RT) 027 was found in animals in North America in the early 2000s (15) but probably moved from animals to humans a decade earlier around the time that RT027 developed resistance to fluoroquinolone antimicrobials (16). This strain was likely to have initially caused infections in the community at a time when community-acquired (CA) CDI [defined as cases with symptom onset in the community or ≤ 48 h after admission to a healthcare facility (17)] was thought infrequent, and diarrhea in the community was rarely investigated. The mutation to fluoroquinolone resistance and high use of fluoroquinolones drove RT027 spread, in North America and later Europe, once it entered the hospital system (16). A similar process now appears to be occurring with *C. difficile* RT078, another animal strain that has increased significantly as a cause of CA-CDI in Europe over the last 10 years (18, 19). *C. difficile* continues to expand in food animal populations, driven by cephalosporin use, and animal strains of *C. difficile* are driving the worldwide increase in CA-CDI.

The whole-genome sequencing era continues to redefine our view of this complex pathogen. The application of high-resolution microbial genomics in a One Health framework (encompassing clinical, veterinary, and environment derived datasets) is the optimal paradigm for advancing our understanding of CDI in humans and animals. This approach has begun to yield critical insights into the genetic diversity, evolution, AMR, and zoonotic potential of *C. difficile*. In this review, evolutionary and zoonotic aspects of CDI are discussed, including the anthropomorphic factors that contribute to the spread of *C. difficile* from the farm to the community.

### Community-Acquired CDI

Surveillance data indicate that CA-CDI comprises a significant fraction of total CDI cases and that the incidence of CA-CDI has been increasing globally (20). In the United States, CA-CDI accounts for around a third of all CDI cases and increased 4-fold during the period 1991–2005 (18, 21–24). In another US study, comparable incidence rates for CA-CDI and hospital-associated CDI (HA-CDI) were reported (11.2 cases/100,000 person-years and 12.1 cases/100,000 person-years, respectively) (18). A recent European multi-center study (97 hospitals in 34 European countries) found 14% of 506 cases were classified CA-CDI (25). In Australia, data from 2011 to 2012 showed CA-CDI accounted for up to a quarter of all cases (26% of 5,109 CDI cases) and has been increasing in recent years (26–28). More recent studies from the USA report higher proportions of CA-CDI around 40% (24). Many studies have noted that individuals with CA-CDI often do not have the “classical” risk factors for CDI acquisition and are generally younger, healthy, and female, without contact with hospitalized patients nor prior antimicrobial exposure (5, 20, 29). In up to 40% of CA-CDI cases, infection is more severe and there are adverse outcomes (hospitalization, treatment failure, complications, colectomy, and recurrence) (19, 30). Notably, *C. difficile* strains acquired in the community can differ in genotype from predominant hospital strains (31), however, *C. difficile* RT078 (see below) has emerged as a significant pathogen associated with both HA- and CA-CDI in the Northern Hemisphere (21, 24, 32–35).

### Zoonotic and Environmental Sources of *C. difficile*

**Figure 1:**
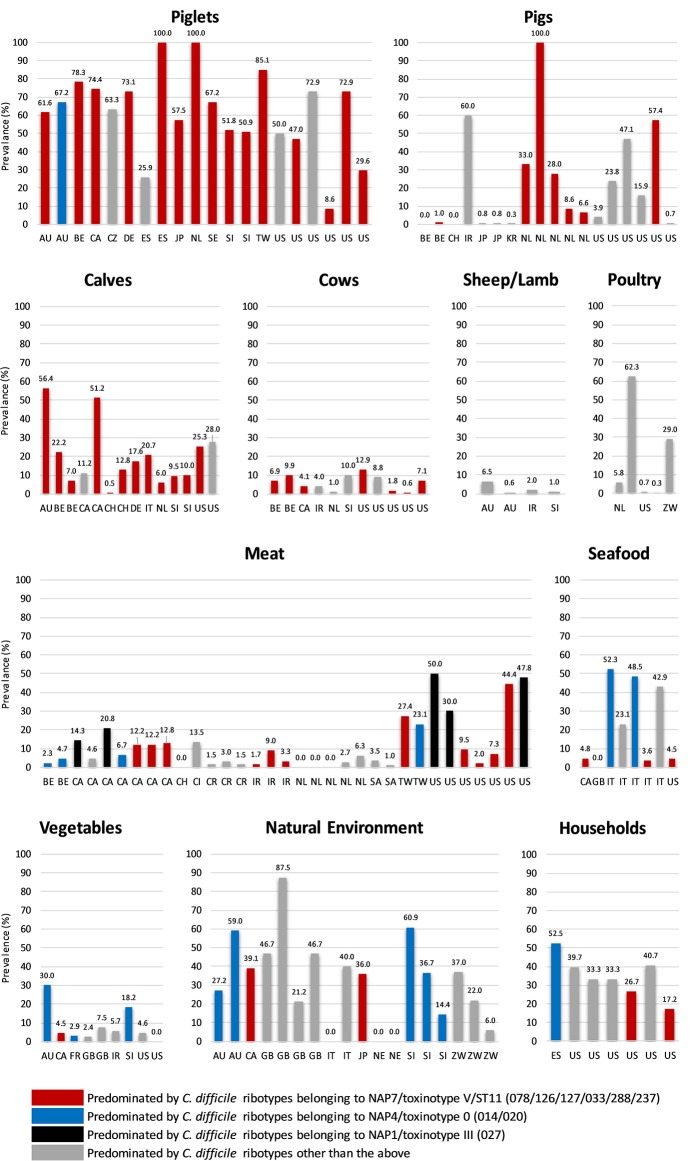
Global prevalence of *C. difficile* in farm animals, food and the environment. Data were taken from 86 studies in 23 countries worldwide (15, 38–122). Categories: Poultry (hens and broilers, Seafood (salmon, perch, clams, shrimp, and mussels), Meat (veal, beef, pork, lamb, chicken, goat, buffalo, and turkey), Vegetables (salads, lettuce, pea sprouts, ginger, carrots, beetroot, potatoes, onions, and spinach), Household (sandbox, shoes, toilet, vacuum, sink, floor), and Natural Environment (compost, lawn, soil, sediment, lake, and river). Two-letter country codes (International Organization for Standardization, ISO): AU, Australia; BE, Belgium; CA, Canada; CH, Switzerland; CI, Ivory Coast; CR, Costa Rica; CZ, Czech Republic; DE, Germany; ES, Spain; FR, France; GB, Great Britain and Northern Ireland; IR, Iran; IT, Italy; JP, Japan; KR, Korea; NE, Nigeria; NL, The Netherlands; SA, Saudi Arabia; SE, Sweden; SI, Slovenia; TW, Taiwan; US, United States of America; ZW, Zimbabwe. NAP, North American Pulse Type. RT027 and all ST11 RTs listed are binary toxin-positive.

*C. difficile* shows remarkable adaption to life within a diverse array of natural and host environments, including its primary habitat the mammalian gastrointestinal tract (as a commensal and/or pathogen), and several secondary habitats such as water, soil, and compost. We have previously reviewed aspects of *C. difficile* prevalence, pathogenicity and antimicrobial resistance (AMR) in non-human reservoirs (36), as have others including excellent reviews by Rodriguez et al. (11) and Candel-Pérez et al. (37). Here we will briefly summarize the key prevalence and molecular data that suggest a zoonotic origin for CDI. Figure 1 summarizes *C. difficile* prevalence data in farm animals, food and the environment taken from 86 studies in 23 countries worldwide (15, 38–122). In many of these studies, differences in *C. difficile* prevalence, strain lineage, toxigenic status, and AMR were identified. These were influenced by a variety of factors including the age of the animal, geographic region, methods used for isolation (e.g., sample type, spore selection, enrichment vs. no enrichment) and veterinary and agricultural practices [see recent reviews (11, 37)].

*C. difficile* is known to colonize numerous food-producing animals including pigs, cattle, sheep, lambs, and poultry. Neonatal animals are viewed as significant reservoirs for *C. difficile* (Figure 1). Prevalence in domestic pigs and piglets averages around ~43%, ranging from 0% [Belgium and Switzerland (98, 103)] to ~50% [USA and Slovenia (61, 70)] and 100% [Spain and The Netherlands (62, 68)]. In cattle and calves, *C. difficile* prevalence averages around 14%, ranging from 0.5% [Switzerland (98)] to ~20% [Italy, Belgium and the USA (43, 46, 103)] to ~50% [Australia and Canada (38, 40)]. On average, a lower prevalence has been reported in ovine hosts [sheep and lambs, ~6% (77)] with prevalence in poultry [hens, broiler chickens] varying considerably [0.3% in the USA (82), to 29.0% in Zimbabwe (83) and 62% in Slovenia (80), mean ~19%]. Due to an absence of colonization resistance afforded by a mature intestinal microflora, during the first weeks of life neonatal pigs and calves are susceptible to disease caused by *C. difficile*. Although data is limited for calves (46) the pathophysiology of CDI in piglets is well-described; diarrhea, dehydration, weight loss, enteritis histologically similar to human lesions, and high mortality (123–125).

Other non-human animal reservoirs of *C. difficile* include cats and dogs (prevalence 0–100%), horses and foals (3–33%) and numerous wild animal species including rabbits, zebra, kangaroos, birds, shrews, Kodiak bears, racoons, camels, donkeys, feral swine, elephants, ibex, molluscs, tamarin monkeys, chimpanzees and, most recently, polar bears (0–100%) (37, 126, 127). The most common *C. difficile* lineage identified in many of these animal studies is multilocus sequence type (MLST, ST) 11, predominated by RT078 and its close relatives RTs 033, 045, 066, 126, 127, and 288 (all binary toxin positive, toxinotype V and cause CDI in humans) (Figure 1). Surprisingly, in Australia, the predominant RT found in pig herds is RT014, one of the most common strains causing CDI in humans worldwide (128) (see below).

*C. difficile* has been recovered from meats and plant-based foods sourced from processing plants, shops, farms and markets throughout Europe, North America and the Middle East (Figure 1). These include retail meat (veal, beef, pork, lamb, chicken, goat, buffalo, and turkey), seafood (salmon, perch, clams, shrimp, and mussels), and salads and vegetables (lettuce, pea sprouts, ginger, carrots, potatoes, onions, and spinach). As is the case with farm animals, the prevalence of *C. difficile* in food varies widely with food type and geographic origin. A high prevalence of *C. difficile* in retail pork, beef, and chicken has been reported in the USA (42%) but studies elsewhere report a much lower prevalence (Taiwan, 23%; Cote d'Ivoire, 14%), especially in Europe (~3.0%) (105, 129, 130). The prevalence of *C. difficile* in seafood varies considerably from ~5.0% in Canada, USA and Wales (99, 108, 118) to ~50.0% in Italy where its presence has been tentatively linked to sewage contamination in local rivers (95). Similarly, the prevalence of *C. difficile* on vegetables varies from 3 to 8% in North America and Europe [ready to eat salads (85, 101, 107, 109, 111, 118)] to 20–56% in Australia [organic beetroot and potatoes (84)] reflecting, possibly, different methods of processing. The molecular epidemiology of *C. difficile* recovered from food largely mirrors that of farm animals (ST11 RTs and common healthcare-associated lineages including 014 and 027, Figure 1).

### Farm to Fork: Agricultural Practices Presenting a Risk for CA-CDI

In its spore form, *C. difficile* persists in various different natural ecosystems [soil, rivers, oceans, lakes, and sediments (114–116, 118, 119)], animals and food (11), and many abiotic environments for example toilets, floors, sinks, and soles of shoes (112, 113, 131). The high transmissibility of the spore (132) combined with its inherent resilience to desiccation, extremes of temperature, and disinfection (133) facilitates the transmission of *C. difficile* between these ecosystems. *C. difficile* spores could be transmitted from the farm environment to humans through a number of mechanisms including direct contact, airborne dispersal, avian, rodent or arthropod vectors (134–137), contamination of meat with feces during slaughter (53, 138) and via animal effluent or effluent by-products such as compost (139). However, CDI is a complex phenomenon encompassing pathogen, host, anthropomorphic and environmental factors, and our understanding of CDI transmission dynamics between production animals and humans is nowhere near perfect. Within Australia, two agricultural practices have been identified which present a credible risk for transmission of *C. difficile* causing CA-CDI: (i) slaughtering of neonatal animals destined for human consumption, and (ii) the recycling of effluent for agricultural purposes such as manufacturing compost which is then disseminated into the community setting (140, 141).

The prevalence of *C. difficile* in Australian veal calves is high although this decreases significantly with increasing age of the animal; 56% from <7-day-old calves, 3.8% in 2–6 month-old calves, and 1.8% in adult cattle (38). The *C. difficile* population within these cattle was dominated by ST11 RTs that all cause disease in humans. Moreover, at slaughter, the prevalence of *C. difficile* in calve feces was 60.0% and a significant proportion of calf carcasses (25.3%) was positive (with a spore concentration of 33 CFU/cm^2^), as a result of spore contamination from gastrointestinal contents during the slaughter process (138). As before, clinically important ST11 RT lineages dominated (138). Australia is one of the very few countries that cull male neonatal dairy calves (veal calves), a practice that exists because they are born male and considered surplus to industry requirements. With *C. difficile* prevalence highest in this neonatal period (127), the unique slaughter age of these animals presents a significant and perhaps under-appreciated risk for contamination of carcasses during the slaughter process. Further, *C. difficile* spores contaminating carcases would likely survive chilling, freezing, and cooking processes (142–145) and may compromise the quality of veal for domestic and export markets. To date, *C. difficile* has not been recovered from retail meat in Australia although only limited surveys have been undertaken mainly on meat from adult animals. Consumer demand for newborn veal in Australia is low and thus there is likely to be limited exposure of consumers to contaminated meats. However, Australia is the third largest beef and veal producer in the world (146), exporting 1.9 million tons of beef and veal per annum to over 100 countries, particularly in Africa, Asia and the Middle East. It is possible that contaminated Australian veal may be contributing to CDI in these regions, however, with the exception of Taiwan where ST11 strains are commonly reported in humans with CDI and farm animals (64, 102, 147), CDI surveillance is lacking in many of these countries. Whatever the level of risk to the domestic and export consumer, it is possible that it can be significantly mitigated by increasing the age that the animal is slaughtered to >3 or more weeks (38).

In the case of Australian piglets and dissemination of the major healthcare-associated lineage RT014, a growing body of evidence points to zoonotic transmission extending from the farrowing shed to the community. First, Australian piglets are major amplification reservoirs for *C. difficile* (67% prevalence nationwide with RT014 comprising 23% of isolates) (52). Second, whilst suckling age piglets are not slaughtered for meat on a large scale, *C. difficile* spores are abundant in treated biosolids, effluent, and piggery wastewater (121, 148–150). These by-products of the pig industry are subsequently recycled to pasture and agriculture for composting and direct irrigation/fertilization of crops and lawn. Third, *C. difficile* has been recovered from 30% of “high-street” retail compost samples in Australia (122), 59% of new roll-on lawn samples in Australia (151) and 20% of various root vegetables from mainstream and organic markets (84). Both lawn and organic vegetables are invariably grown in compost/soil containing animal manure. In these studies, RT014 comprised 7, 39, and 10% of isolates, respectively. Finally, the use of potent, late generation cephalosporins in human and veterinary medicine is a major driver of (i) *C. difficile* colonization and onset of disease in pigs; (ii) amplification and persistence of *C. difficile* in piggeries; (iii) spill-over of spores into the environment; and (iv) onset of CDI in the community (135, 140, 141).

## Genomic Insights Into The Evolution And Transmission Of *C. Difficile* In Animals And Humans

### Microevolution in the *C. difficile* Core Genome

The next generation sequencing era has seen the development of exquisitely sensitive, cost-effective, and rapid, benchtop whole-genome sequencing (WGS) technologies. Combined with new WGS-based genotyping tools, these technologies are shaping the future of infectious diseases surveillance. Core genome single nucleotide variant (SNV) analysis is an ultra-fine scale discriminatory method that uses WGS to detect transmission and outbreaks of bacterial pathogens (152, 153). SNV analysis is restricted to the non-repetitive, non-recombinative core genome which contains essential genes common to all isolates under analysis that are often vertically inherited and most likely to have the strongest signal-to-noise ratio for inferring phylogeny (152, 153). For *C. difficile*, SNV analysis uses a fixed-rate molecular clock derived from serial isolation of strains from clinical cases, estimated to be in the region of 1.47 × 10^−7^ to 5.33 × 10^−7^ mutations per site per year, to identify signatures of plausible clonal transmission (154, 155). This equates to 1–2 SNVs per genome per year. For studies of *C. difficile* transmission, a clonal group is therefore defined as two or more strains differing by <2 SNVs in their core genome, with ≥10 SNVs used as a threshold for genetically distinct isolates (154–157). For longer-term ecological studies, these thresholds may not hold true as the genetically quiescent nature of *C. difficile* spores may result in underestimating the evolutionary distance between strains (19).

The ultra-fine scale resolution of this technique is superior to conventional *C. difficile* typing methods including PCR ribotyping, pulsed-field gel electrophoresis (PFGE), MLST, Rep-PCR, toxinotyping, and amplified fragment-length polymorphism (AFLP) fingerprinting (152). It also shows discriminatory power comparable, and in some cases superior, to multilocus variable-number tandem repeat analysis (MLVA) (152, 157) and the recently developed core genome MLST scheme (158). Supplementary Table 1 provides a summary of bioinformatics tools and algorithms involved in a *C. difficile* SNV pipeline.

For *C. difficile*, SNV-based typing has been used to study the microevolution of CDI in the hospital setting (154) and to investigate localized transmission and international dissemination of major clinically important lineages such as RTs 027 (16) and 017 (159). But as outlined below, this approach has also been used to delineate cryptic transmission pathways of *C. difficile* between animals, humans, and their shared environment. In doing so, these genomic studies have redefined our understanding of the ecology and evolution of this complex species.

### *C. difficile* RT078

**Figure 2:**
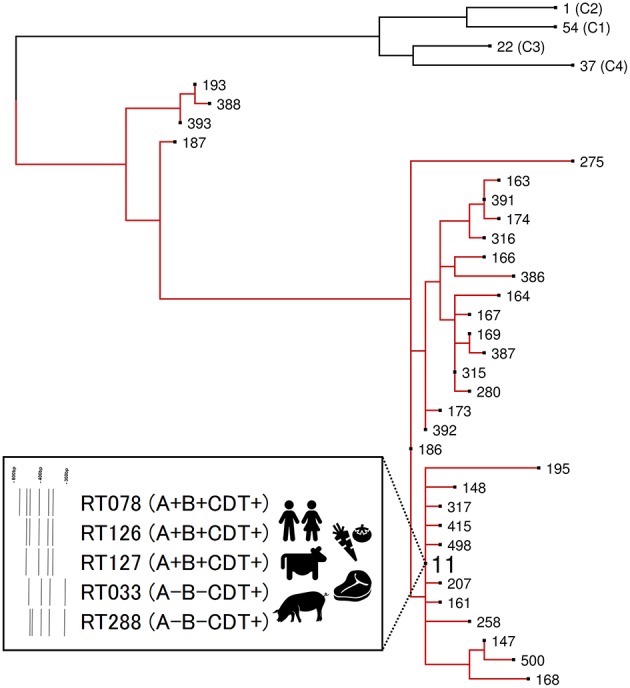
Evolutionary clade 5. Maximum-likelihood phylogeny based on concatenated MLST allele sequences (seven loci, 3,501 bp) for 32 known clade 5 STs. For global phylogenetic context, well-characterized representatives of major MLST clades C1 (ST54, RT012), C2 (ST1, RT027), C3 (ST22, RT023), and C4 (ST37, RT017) are also shown. Branches for clade 5 are shown in red. The inset box highlights major ST11 RT sublineages of clinical and agricultural/livestock importance with associated toxin gene profiles and graphical representation of 16s−23s rRNA PCR ribotyping banding patterns.

*C. difficile* RT078 belongs to evolutionary clade 5 and is the principal ST11 sublineage (Figure 2). Between 2005 and 2008, RT078 rose from 11th to become the 3rd most frequently encountered RT in European hospitals (25), an increase particularly evident in the Netherlands where, from 2005 to 2008, Dutch hospitals would see the total prevalence of RT078-associated cases increase from 3 to 13% (32). These RT078 cases of CDI were in younger patients and with community-onset (32, 33). Comparable rates have been found in North America (21, 24, 35) with one study reporting 46% of all RT078 isolates were community acquired (160). As with many toxigenic *C. difficile* RTs, RT078 can be carried asymptomatically (161, 162). *C. difficile* RT078 has established significant reservoirs in North American, European, and Asian pigs and cattle and is often reported as the dominant type irrespective of age, diarrheal status or other farm-specific factors (37, 127). In an important Dutch study of *C. difficile* spore acquisition, Hopman et al. (68) demonstrated that piglets delivered by cesarean-section were *C. difficile*-negative yet were rapidly colonized with *C. difficile* RT078 spores within 48 h.

The virulence potential of RT078 has been likened to that of epidemic RT027 with which it shares similar genetic features. These include the major virulence genes *tcdA, tcdB*, and *cdtA/B* involved in toxin production, and an aberrant toxin regulator gene *tcdC* (deletions, nonsense mutations, and premature stop codons) leading to a reduction in log phase repression of toxin expression. The role for the latter in the observed hyper-virulent disease phenotype seen also in RT078 infections i.e., more toxin, increased mortality and morbidity, remains speculative (32, 163–167). *C. difficile* RT078 strains are often multidrug-resistant (MDR) (161, 168) and, compared to other RTs, including RT027, show remarkable resilience to extremes of temperature (80 to 96°C) and water treatment processes (142, 143, 145). It has also been proposed that the emergence and global dissemination of RT078 in humans is linked to an enhanced ability to metabolize the food additive trehalose (169). These virulence and survival traits may explain the successful dissemination of this lineage in production animals and humans worldwide. Unsurprisingly, it has received major attention as a potentially zoonotic lineage.

### Zoonotic Transmission of *C. difficile* RT078 Between Humans and Animals

Genetic studies using MLST, MLVA, Rep-PCR and AFLP fingerprinting have all provided significantly higher strain resolution of RT078 populations compared to conventional PCR ribotyping. In 2010, Bakker et al. (170) found 85% of RT078 isolates of human and porcine origin in the Netherlands were genetically related and, in many instances, indistinguishable by high-resolution MLVA. In 2012, Stabler et al. (171) used MLST to analyse 385 *C. difficile* isolates from different geographical locations (Europe, North America, and Australia) and sources (human, food, and animal). Strains of RT078 from humans, food and animals, some from different countries and continents, were indistinguishable (all sharing seven identical housekeeping genes, ST11) (171). More recent work from Taiwan showed RT078 isolated from pig farms shared identical Rep-PCR fingerprints as RT078 strains derived from humans with CDI in hospitals in the same region (64). Similarly, in Spain, RT078 of human and animal origin were clustered together by AFLP (172). Evidence from Japan suggests RT078 has been introduced from Europe. Usai et al. found Japanese pig RT078 strains clustered (by MLVA) with European human and pig RT078 strains (86), and Niwa et al. found a single MLVA cluster of RT078 responsible for five cases of colitis in Japanese racehorses (173). Both pigs and racehorses are internationally traded in Japan; thus, RT078 may have been imported into Japan from Europe via live animals.

Natural and diverse reservoirs of RT078 support the hypothesis that CDI may have a zoonotic origin. To date, a few key WGS-based studies have led to significant advancement in understanding the true zoonotic potential and evolution of the RT078 and its close relatives. In 2013, Knetsch et al. (161) used core genome SNV typing to compare 65 *C. difficile* RT078 isolates of human and porcine origin sourced over a 10-year period in The Netherlands. Using Bayesian techniques, an RT078 population-specific mutation rate was estimated to be 2.72 × 10^−7^ substitutions per site per year, equating to around 1 SNV per genome per year—a figure comparable with earlier estimates (154, 155). A core genome phylogeny showed isolates of human and porcine origin clustering together. Notably, the analysis showed a pair of human and pig isolates from the same pig farm in The Netherlands to be indistinguishable (zero SNVs difference in their core genome). Working in pig husbandry or living in (or visiting) areas with a high density of pigs increased the risk of acquiring *C. difficile* due to exposure to pig feces (161). Whilst the transmission of RT078 between a pig and pig farmer within the confines of a pig-rearing facility might not be that surprising, it was nonetheless the first ever confirmation that interspecies transmission of *C. difficile* had occurred (161). The exact mode of transmission between these species remains unclear. Whilst these data appear to support the theory that CDI is a zoonosis, a common environmental source, asymptomatic carriage and/or zooanthroponotic (human to animal) transmission cannot be ruled out.

In 2017, the same authors (174) extended these findings. They investigated microevolution in the core genome of 248 *C. difficile* RT078 strains sourced from humans and animals in 22 countries. This study provided the first estimate of the global RT078 population structure and yielded new insights into the potential and extent for zoonotic spread. Extensive clustering of *C. difficile* RT078 from human clinical cases and food animals was observed, with clear instances of interspecies clonal transmission, only this time, the significant clustering of clones supported evidence of bidirectional spread of *C. difficile* RT078 between production animals and humans. Moreover, there was only limited geographic clustering with clones of *C. difficile* RT078 spread multiple times across multiple towns, countries and continents, in particular between North America and Europe: one example was the transmission of an RT078 clone between an animal in Canada and humans in the United Kingdom. This indicated interspecies transmission of *C. difficile* RT078 was not restricted to a local population of humans and production animals, as previously shown in the 2014 Dutch study. Together, these data revealed a highly linked intercontinental transmission network of *C. difficile* RT078 between humans and animals and provided further evidence that CDI has a significant zoonotic component (174). Yet it also showed that, in contrast to another classic enteric pathogen *Salmonella enterica* which has distinct animal- and human-associated populations, *C. difficile* RT078 appeared to be a clonal population moving frequently (and likely over long time periods) between production animal and human hosts, with no geographical constraints.

### ST11 Is a Heterogeneous Lineage of Major One Health Importance

ST11 is an ancient evolutionary lineage comprising at least a dozen CDT^+^ ribotypes that cause CDI in humans with significant ecological niches in production animals worldwide (175) (Figure 2). As is apparent, and for good reasons, there has been a strong focus on the ST11 sub-lineage RT078, however, until recently, little was known about the evolutionary history and zoonotic potential of other ST11 RTs. Our recent study (175) addressed this knowledge gap, using WGS to investigate population structure and clonal transmission in over 200 strains of major ST11 RTs 078, 126, 127, 033, and 288 sourced from human and veterinary/environmental origin across Australia, Asia, Europe, and North America. A core genome phylogeny showed the global ST11 population structure largely mirrored RT sub-lineage, with discrete evolutionary clusters congruent with RTs 078/126, 127, 033/288. Core genome SNV analysis found multiple instances of inter- and intra-species clonal transmission in all RT sub-lineages. Interspecies clonal groups comprised *C. difficile* isolates derived from health care facilities and farm animals spread across different states, countries, and continents, often without any healthcare association. Our findings independently confirm and extend the work of Knetsch et al. (161, 174) revealing a globally-disseminated network of *C. difficile* ST11 clones with the capability and proclivity for reciprocal zoonotic and/or anthroponotic transmission. Moreover, this study showed for the first time that non-RT078 ST11 strains such as RTs 126, 127, 033, and 288 also display a high zoonotic potential and should also be considered lineages of emerging One Health importance.

### Antimicrobial Resistance and ST11 Evolution

Antimicrobials are a crucial component in the pathogenesis of CDI; they play a central role in the establishment of infection and, paradoxically, remain the preferred option for treatment (176).

AMR is, therefore, a key factor driving epidemiological changes in CDI (1). As we have seen with virulent *C. difficile* RT027 epidemic lineage, outbreaks emerge when the inherent resistance of *C. difficile* to cephalosporins is combined with acquired resistance to high-risk antimicrobials known to incite CDI, such as fluoroquinolones (16). In all the above WGS-based studies of RT078 and ST11, substantial AMR repertoires were identified. In the Dutch study (161), interspersed throughout the RT078 phylogeny were clones common to humans and livestock harboring identical mobile genetic elements (MGEs) conferring resistance to streptomycin (Tn*6235, aphA1*^+^) and tetracycline (Tn*6190, tet*M^+^) (161). In the later study by Knetsch et al. (174), the global population of RT078 contained a broad array of AMR genes encoding resistance to aminoglycosides and streptothricin (*aph3*′*-III, ant6*′*-Ib, Sat4A*), erythromycin (*erm*B^+^), and tetracycline (*tetM, tetO, tet32, tet40, tet44*). The gene *cdeA* encoding a multidrug efflux transporter was found in all isolates (174).

In our ST11 study (175), half of all strains showed phenotypic resistance to one or more of tetracycline, moxifloxacin, erythromycin, and clindamycin, of which a quarter, predominantly RTs 126/078, were resistant to ≥3 of these agents. Underscoring this resistance was an array of AMR genetic loci including chromosomal mutations in *gyrA/B* (fluoroquinolone resistance) and MGEs conferring resistance to macrolides and lincosamides (Tn*6194*; *ermB*^+^), and tetracycline (Tn*6190*; *tet*M^+^ and Tn*6164*; *tet44*^+^), the latter a 106 kb genetic island apparently specific to RT078 (177). This was the first such report of Tn*6194* from animals in the world. This element is the most common *ermB*-containing element found in human clinical isolates in Europe and is a defining genetic feature of epidemic RT027 (16, 178, 179). A phenotypically silent *vanB2* transposon (likely from *Enterococcus faecalis*) was also found in a *C. difficile* RT033 strain isolated from an Australian veal calf at slaughter (180). Another common ruminant species *Erysipelothrix rhusiopathiae* appeared to be the origin of the numerous aminoglycoside resistance gene clusters present in all ST11 sub-lineages.

In a compelling new study, Dingle et al. (181) present a strong case for antimicrobial selection influencing the recent evolutionary history of *C. difficile* RT078. A time-scaled phylogeny built from the core genome of over 400 international *C. difficile* RT078 strains revealed three major clonal expansions (a rapid, recent international spread of RT078 clones). Two-thirds of all RT078 were tetracycline resistant. Remarkably, a common ancestor of each clonal expansion had independently evolved tetracycline resistance via the acquisition of distinct *tetM* alleles carried on closely related Tn*916-like* elements, an analogous situation to the emergence of fluoroquinolone resistance in RT027 (16). The parallel *tetM* associated clonal expansions were estimated to have occurred sometime around the year 2000, at a time when the number of RT078-associated clinical cases (at least in Europe) started to increase. Moreover, the three *tetM* alleles show significant homology (97–100% sequence identity) with *tetM* genes belonging to established zoonotic species such as *E. faecalis, Escherichia coli*, and *Streptococcus suis*—further supporting an agricultural origin for RT078. The authors note that *S. suis* has striking parallels with *C. difficile* RT078—it is a globally disseminated human pathogen which has established substantial reservoirs in pigs and has displayed recent increases in tetracycline resistance (182, 183). In summary, these phylogenetic data are consistent with an evolutionary response to tetracycline selective pressure. The inappropriate and overuse of tetracycline in animal husbandry is well-recognized (184). This selective pressure, combined with the rapid, international spread of *C. difficile* RT078 via the food chain and other agricultural vectors provides a plausible explanation for the clinical prominence of this lineage in humans.

### Interspecies Transfer of *C. difficile* RT014 Between Humans and Animals

*C. difficile* RT014 is a toxigenic (A^+^B^+^CDT^−^) and highly successful lineage of *C. difficile* belonging to MLST clade 1. RT014 is consistently among the most common RTs causing CDI in European healthcare systems, and in Australia it has been the most prevalent RT causing human infection for many years, accounting for ~25% of all CDI cases (10, 185–188). The zoonotic potential of this RT was initially thought to be quite low as its prevalence in production animals in Europe was low and it was absent from livestock in Asia. In Australia, there was a completely different and intriguing story. In 2013, a nationwide cross-sectional study of *C. difficile* in 21 pig farms in Australia found RT014 to be the most prevalent RT in neonatal pigs aged <14 days, accounting for 23% (*n* = 26/154) of isolates (52). With rates of CDI in Australia increasing markedly in recent years (24% in 2011–2012 alone) and a significant rise in CA-CDI (26), the establishment of significant RT014 reservoirs in porcine populations in Australia suggests zoonotic transmission as a plausible source of human infection.

**Figure 3:**
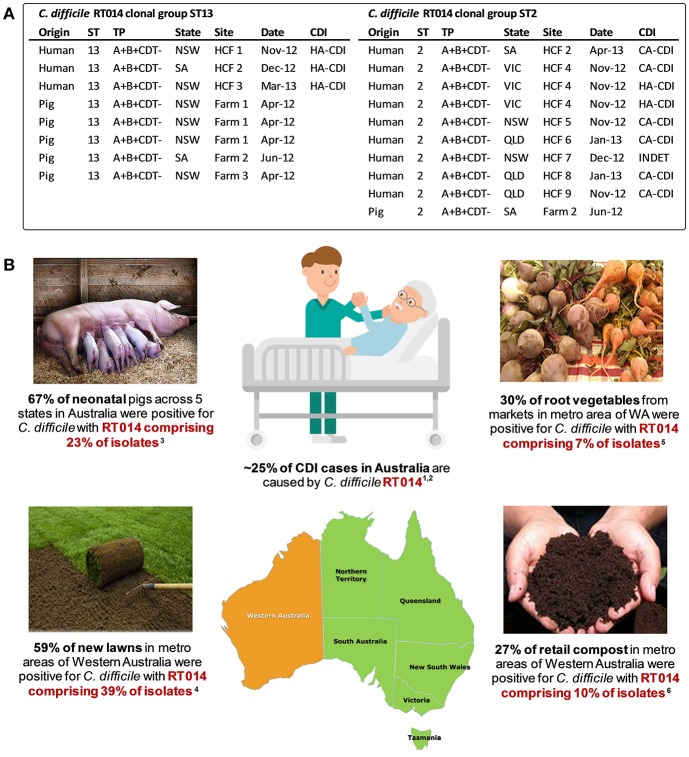
Transmission networks and community reservoirs of *C. difficile* RT014 in Australia. **(A)** summary of ST13 (*n* = 8) and ST2 (n = 10) RT014 clonal groups found in pigs and humans with CDI in Australia, adapted from Knight et al. (128). A clonal group is defined as two or more strains differing by <2 SNVs in their core genome. HCF, healthcare facility; NSW, New South Wales; SA, South Australia; VIC, Victoria; QLD, Queensland; INDET, indeterminate. **(B)** summary of RT014 ecological niches in Australia. ^1^Knight et al. (186); ^2^Collins et al. (188); ^3^Knight et al. (52); ^4^Moono et al. (151); ^5^Lim et al. (84); ^6^Lim et al. (122).

To examine the true extent of genetic relatedness, a collection of 40 contemporaneous isolates of RT014 of human and porcine origin in Australia were subjected to WGS (128). A total of three distinct STs were identified in this RT014 collection (STs 2, 13, and 49), and in each, human and porcine populations were intermingled, signaling a very recent shared ancestry. A phylogeny based on evolution in 1,260 core orthologous genes (1,019,160 bp, ~25% of bases in an average *C. difficile* genome) showed geographically and temporally unconstrained clustering of human and animal *C. difficile* RT014 strains in all three STs again supporting a close genetic relationship. Finally, a phylogeny-based on evolution in non-recombinant 1,287 core genome SNVs provided ultra-fine scale resolution of the RT014 population, identifying multiple instances of plausible interspecies clonal transmission. In total, 42% of *C. difficile* RT014 strains from humans with CDI showed a clonal relationship (differing by no more than two SNVs in their core genome) with one or more RT014 strains derived from pigs. Remarkably, many RT014 clones originated from pigs and humans in states separated by thousands of kilometers, collected many months apart, and half of the human isolates in these clonal groups originated from cases classified as CA-CDI, representing the acquisition of CDI outside of the hospital system (Figure 3). Long range transmission of *C. difficile* RT014 clones suggests direct contact between humans and colonized livestock is perhaps unlikely, and there was no evidence here. Given what we know of the *C. difficile* colonization-transmission cycle in the farrowing environment and wider livestock industry, it is conceivable that over an extended period there has been frequent long-range indirect interspecies transmission through human exposure to contaminated retail meat but more likely contaminated piggery by-products such as manure and compost in the community setting (Figure 3). Indeed, genomic studies from the USA and Europe have shown that the household environment and pet dogs are colonized with *C. difficile* RT014/ST2 representing reservoirs of RT014 in the community (124, 125).

### The *C. difficile* Pan-Genome: Insights Into the Ecology of a Complex Pathogen

A bacterial pan-genome describes the full complement of genes in a species or individual phylogenetic lineage. It comprises a core component (those genes present in all strains) and an accessory or adaptive component (genes absent from one or more strain or unique to a particular strain) (189). Early microarray-based studies estimated the *C. difficile* pan-genome to be comprised of 9,640 coding sequences (CDS) with a core genome component many orders of magnitude lower at 600–3,000 CDS (190–192). More recent WGS based studies of RT014 (128), RT078 (174), and ST11 (175) from humans and animals have provided further insights into the genetic diversity, plasticity and ecology of zoonotic *C. difficile* lineages.

Analysis of 44 Australian RT014 genomes (STs 2, 13, and 49) revealed a large pangenome (7,587 genes) comprising a core genome of 2,296 genes (30.3% of the total gene repertoire) and an accessory genome of 5,291 genes (128). Moreover, the human and porcine populations shared near identical proteomes (128). The global RT078 population (248 genomes from four continents) possessed a large pangenome of 6,239 genes with a core genome of 3,368 genes (53.9% of the total gene repertoire) and an accessory genome of 2,871 genes (174). Finally, the global ST11 population (207 genomes from four continents including RTs 078, 126, 127, 033, and 288) was defined by a massive pan-genome (10,378 genes), a remarkably small core genome of 2,058 genes (only 19.8% of the gene pool) and an accessory genome of 8,320 genes (175). In the case of RT014 and ST11, power-law regression analysis determined the pangenomes to be “open,” that is, size increases indefinitely when adding new genomes. For example, in the ST11 analysis, after sequencing over 200 genomes there is an average of 16 new genes contributed to the gene pool with each additional sequenced strain (175).

The size and openness of a pan-genome is also a very useful proxy for characterizing the lifestyle of a bacterial species (193). The pan-genome data derived from these zoonotic and agricultural-associated *C. difficile* lineages predict a species with a sympatric lifestyle, occupying niches in extremely diverse environments that are enriched with mixed microbial communities of prokarya and archea (193). This is true of *C. difficile*, a versatile species which shows extraordinary adaption to multiple ecosystems including the gastrointestinal tract of multiple mammalian hosts, and several secondary habitats such as water, soil, and composts and invertebrate species (179). In contrast to allopatric and intracellular species such as *Rickettsia rickettsii* and *Chlamydia trachomatis*, which have small closed pan-genomes and live in isolated niches with limited exchange with the global microbial gene pool, sympatric species like *C. difficile* (and *C. botulinum*) have larger, more complex open pan-genomes. Sympatry also means a higher frequency of gene acquisition events and a higher probability of acquiring parasitic DNA i.e., transposons and bacteriophages, both contributing to an increase in pan-genome size (193, 194). Indeed, underscoring the substantial genetic diversity in these zoonotic *C. difficile* lineages were large and diverse collections of clinically important prophages of the *Siphoviridae* and *Myoviridae* (128, 175) and AMR genetic elements (128, 174, 175). As corroborated by Dingle et al. in RT078 (181), many of these underlying AMR elements show evolutionary origins in commensal species residing within the gut of farm animals. Examples being macrolide resistance genes from *Campylobacter coli* (cryptic), aminoglycoside, and streptothricin genes cassettes from *E. rhusiopathiae*, and a plethora of tetracycline resistance genes from *S. suis, E. faecalis, Megasphaera elsdenii, C. jejuni, and C. perfringens* (128, 161, 174, 175). Moreover, AMR elements Tn*6194* (*erm*B^+^) and Tn*5397* (*tet*M^+^) are capable of intra-species transfer to different *C. difficile* RTs and even inter-species transfer to other genera (16, 191, 195).

Together, the phylogenetic, pangenome, and AMR data show that these zoonotic *C. difficile* lineages have the capability and propensity to move between humans, production animals, and their shared environment. By occupying niches within multiple host species, these *C. difficile* lineages are able to access and exchange DNA with an enormously diverse metagenome, particularly the ruminant gut and soil microbiota. Such promiscuous behavior provides *C. difficile* with a potential selective advantage over taxa inhabiting the same gut ecosystem, be it the pig, cow or human intestinal tract, therefore greatly enhancing their ability to adapt to fluctuating environmental factors and their likelihood of success.

Finally, in the case of ST11, it is remarkable that even after sampling >200 ST11 strains from over a dozen unique RT sub-lineages spread over four different continents; the complete gene complement of this lineage was not captured (175). With over 420 STs and >600 RTs currently recognized, it is likely that the complete species pan-genome for *C. difficile* could be astonishingly high. Such enormous diversity is more typical for phylogenetic distances between genera within a family, rather than strains within a species (179). In light of recent calls for taxonomic revisions (196–199), it is possible that *C. difficile* may, in fact, be a complex of sub-species divided along the major evolutionary clades.

## Future Directions And Challenges

The One Health paradigm is a philosophical approach to improving and safeguarding the health of humans, animals and the environment and, importantly, recognizes that these three areas are inter-related (200). Specifically, improved treatment of disease common to humans and animals can be achieved through the application of interdisciplinary approaches between human and veterinary medicine, and the analysis of environment-derived isolate datasets. In this regard, CDI is the quintessential One Health issue (141). As we have highlighted here, the application of high-resolution microbial genomics in a One Health framework is the optimal paradigm for advancing our understanding of CDI in humans and animals. Together, this body of evidence challenges the existing paradigm and long-held conception that CDI is primarily a healthcare-associated infection and provides compelling evidence that CDI has a significant zoonotic component. More important, these findings should stimulate new discussions about One Health focused interventions for CDI.

Collaboration between human and veterinary medicine will be essential if we are to safeguard the health of humans and production animals (141). First and foremost, measures which reduce the levels of *C. difficile* spores in the piggery environment are of paramount importance, not only for mitigating the risk of community acquisition but also for improving animal health (141). In human medicine, these measures comprise stringent infection control policies such as case isolation, reduced use of late-generation cephalosporins, hand hygiene and deep environmental cleaning (201, 202). Analogous interventions have been employed in the veterinary hospital setting with a significant reduction in CDI cases (203); however, the vast scale of modern production animal systems may hinder successful implementation. Also, the frequent disagreement between clinicians, veterinarians and the livestock industry regarding appropriate risk management of *C. difficile* in animal populations remains an additional, significant hurdle to overcome (141, 204).

With several candidate *C. difficile* vaccines in development (205), immunization of livestock could be a highly effective way to reduce the overall prevalence of *C. difficile* and is a good example of an integrative One Health approach to tackling CDI (141). Finally, continued genetic and phenotypic surveillance of *C. difficile* is critical to an enhanced understanding of epidemiological and genetic factors contributing to the emergence, evolution, and spread of CDI (152, 179). Crucially, if we are to identify improved infection prevention and control strategies, and public health interventions designed to mitigate the risk of *C. difficile* transmission, it is imperative that such studies should have a strong One Health focus by including analysis of *C. difficile* strains derived from humans, animals and food, and their shared environment. As much of the focus to date has been on the ST11 group and RT014, future studies should examine the potential for clonal relationships between other lineages circulating in clinical and animal/environmental settings. As illustrated by the studies highlighted in this review, WGS will play a central role in this, providing a level of discrimination far beyond that achievable by conventional molecular typing methodologies.

## Author Contributions

All authors listed have made equal intellectual contribution to the work, and approved it for publication.

### Conflict of Interest Statement

The authors declare that the research was conducted in the absence of any commercial or financial relationships that could be construed as a potential conflict of interest.
