# Supplementary material for: Genomic Delineation of Zoonotic Origins of Clostridium difficile
Source: Front Public Health. 2019 Jun 20;7:164. doi: 10.3389/fpubh.2019.00164 (PMC6595230; doi:10.3389/fpubh.2019.00164)
Supplement: Supplementary file 1 [file Table_1.DOCX]

# Supplementary Table 1

| **Step** | **Bioinformatics tool** | **Reference** |
| --- | --- | --- |
| Reference mapping | Smalt | sanger.ac.uk/science/tools/smalt-0 |
|  | Bowtie 2 | (1) |
|  | Stampy | (2) |
|  | BWA | bio-bwa.sourceforge.net/ |
| Processing/quality control | SAMtools | (3) |
|  | BBmap | (4) |
|  | Qualimap | (5) |
| Variant calling | SAMtools | (3) |
|  | BCFtools | (3) |
| Variant filtering: quality, depth, and heterozygosity | SNPEff | (6) |
|  | Grep | gnu.org/s/grep/ |
| Variant filtering: repetitive regions and indels | SNPEff | (6) |
|  | Vcftools | (7) |
|  | Blastn | (8) |
|  | Dustmasker | (9) |
| Variant filtering: recombinative regions | Vcftools | (7) |
|  | Gubbins | (10) |
|  | ClonalFrame | xavierdidelot.github.io/clonalframe |
|  | SNPEff | (6) |
| Phylogenetics | RAxML | (11) |
|  | ClonalFrameML | (12) |
|  | BEAST2 | (13) |
|  | Figtree | (14) |
|  | iToL | (15) |
| Variant annotation and pairwise SNV analysis | SNPEff | (6) |
|  | Python | github.com/python |

**References**

1. Langmead B, Salzberg SL. Fast gapped-read alignment with Bowtie 2. Nat Methods. 2012;9:357-9.

2. Lunter G, Goodson M. Stampy: a statistical algorithm for sensitive and fast mapping of Illumina sequence reads. Genome Res. 2011;21:936-9.

3. Li H, Handsaker B, Wysoker A, Fennell T, Ruan J, Homer N, et al. The sequence alignment map format and SAMtools. Bioinformatics. 2009;25:2078-9.

4. Bushnell B. BBMap short read aligner. Web Citation: <http://sourceforgenet/projects/bbmap>. 2015.

5. Okonechnikov K, Conesa A, Garcia-Alcalde F. Qualimap 2: advanced multi-sample quality control for high-throughput sequencing data. Bioinformatics. 2015;32:292-4.

6. Cingolani P, Platts A, Wang le L, Coon M, Nguyen T, Wang L, et al. A program for annotating and predicting the effects of single nucleotide polymorphisms, SnpEff: SNPs in the genome of *Drosophila melanogaster* strain w1118. Fly. 2012;6:80-92.

7. Danecek P, Auton A, Abecasis G, Albers CA, Banks E, DePristo MA, et al. The variant call format and VCFtools. Bioinformatics. 2011;27:2156-8.

8. Morgulis A, Gertz EM, Schaffer AA, Agarwala R. A fast and symmetric DUST implementation to mask low-complexity DNA sequences. J Comput Biol. 2006;13:1028-40.

9. (APIQ) APIQAP. Australian Pork Industry Quality Assurance Program (APIQ) - Implementation Manual V3.33. Australian Pork Industry Quality Assurance Program (APIQ) - Implementation Manual. 2013.

10. Croucher NJ, Page AJ, Connor TR, Delaney AJ, Keane JA, Bentley SD, et al. Rapid phylogenetic analysis of large samples of recombinant bacterial whole genome sequences using Gubbins. Nucleic Acids Res. 2015;43:e15.

11. Stamatakis A. RAxML-VI-HPC: maximum likelihood-based phylogenetic analyses with thousands of taxa and mixed models. Bioinformatics. 2006;22:2688-90.

12. Didelot X, Wilson DJ. ClonalFrameML: efficient inference of recombination in whole bacterial genomes. PLoS Comput Biol. 2015;11:e1004041.

13. Bouckaert R, Heled J, Kuhnert D, Vaughan T, Wu CH, Xie D, et al. BEAST 2: a software platform for Bayesian evolutionary analysis. PLoS Comput Biol. 2014;10:e1003537.

14. Rambaut A. FigTree, a graphical viewer of phylogenetic trees. Web Citation: <http://tree.bio.ed.ac.uk/software/figtree>. 2007.

15. Letunic I, Bork P. Interactive tree of life (iTOL) v3: an online tool for the display and annotation of phylogenetic and other trees. Nucleic acids research. 2016;44(W1):W242-W5.
